# RAE1 promotes BMAL1 shuttling and regulates degradation and activity of CLOCK: BMAL1 heterodimer

**DOI:** 10.1038/s41419-019-1346-2

**Published:** 2019-01-25

**Authors:** Xulei Zheng, Xu Zhao, Yingying Zhang, Hao Tan, Bojun Qiu, Tengjiao Ma, Jiarong Zeng, Dachang Tao, Yunqiang Liu, Yilu Lu, Yongxin Ma

**Affiliations:** Department of Medical Genetics, State Key Laboratory of Biotherapy, West China Hospital, Sichuan University and Collaborative Innovation Center, 610041 Chengdu, China

## Abstract

Circadian rhythm is an autoregulatory rhythm, which is sustained by various mechanisms. The nucleocytoplasmic shuttling of BMAL1 is essential for CLOCK translocation between cytoplasm and nucleus and maintenance of the correct pace of the circadian clock. Here we showed that RAE1 and NUP98 can promote the degradation of BMAL1 and CLOCK. Knockdown of RAE1 and NUP98 suppressed BMAL1 shuttling, leading to cytoplasm accumulation of CLOCK. Furthermore, Chip assay showed that knockdown of RAE1 and NUP98 can enhance the interaction between CLOCK: BMAL1 and E-box region in the promoters of Per2 and Cry1 while reducing its transcription activation activity. Our present study firstly revealed that RAE1 and NUP98 are critical regulators for BMAL1 shuttling.

## Introduction

Circadian rhythm is a biological rhythm governing physiology and behavior with a period of 24 h that runs in tight synchrony with environmental cues, such as light and temperature^1^. It is ubiquitous and evolutionarily conserved in species from archaebacteria to humans^2^. At the molecular level, circadian clocks are based on cell-autonomous and autoregulatory rhythm, which is generated by transcription–translation feedback loops^3,4^. In this model, the heterodimeric transcriptional activators BMAL1 and CLOCK that contain bHLH and PAS domains promote the transcription of CACGTG E-box or E-box-like containing clock-controlled genes (CCGs), such as Cryptochrome (Cry1-2) and Period (Per1-3) genes^5–7^. CRY and PER proteins are synthesized in the cytoplasm and enter nucleus to bind and inhibit BMAL1: CLOCK heterodimers^2,8,9^. Besides, two nuclear receptors ROR and REV-ERB are involved in the BMAL1 transcription regulatory loops^10–12^. Posttranslational modifications and proteolysis of the clock proteins are involved in the regulation of circadian clock^1,13^.

The clock proteins enter nucleus to perform functions. So the translocation between cytoplasm and nucleus is critical in maintaining the correct pace of the circadian clock. Several clock proteins have been shown to contain nuclear localization signal (NLS) sequences, such as BMAL1, PER, REV-ERB, etc^14–16^. Besides, BMAL1 and PER2 contain nuclear export signal (NES) sequences. But the sequence of NLS and NES is not found in CLOCK^14,16^. Interestingly, PER2 shuttles in nucleocytoplasm and carries CRY entering nucleus, while the CRY blocks nuclear export of PER2 reversely^17^. Throughout the circadian cycle, BMAL1 mRNA and protein levels in the SCN and other peripheral clock cells oscillate robustly, whereas CLOCK is constitutively expressed, and the abundance of CLOCK was in molar excess of BMAL1^13,15,18,19^. The CLOCK: BMAL1 complex enter nucleus by BMAL1-dependent shuttling, and the shuttling of BMAL1 dynamically control transcription activation activity and proteolysis of the CLOCK: BMAL1 heterodimers^14,20^. But the regulating mechanisms of BMAL1 shuttling involved especially in the nucleus remain to be elucidated.

The mRNA export factor (RAE1) (also named Gle2 or Mnrp41), a conserved WD40 proteins, is homologous of BUB3^21^. RAE1 and BUB3 play essential, overlapping, and cooperating roles in the mitotic checkpoints^22^. Previous studies suggested that RAE1 is involved in the mRNA export pathway, while not the only way in mammals^22,23^. RAE1 binds to GLEBS motif of the nucleoporin NUP98 to function together. They are the 2 of about 30 different proteins found in the nuclear pore complex, and their interaction can contribute to mRNA export^24,25^. Besides, RAE1 and NUP98 form a complex with Cdh1-activated antigen-presenting cell anaphase-promoting complex (APC) to delay APC-mediated ubiquitination of SECURIN to maintain the mitotic checkpoint, and the RAE1 and NUP98 function in spindle assembly to prevent chromosome missegregation^26–28^. RAE1 and NUP98 play an indispensable role in cell cycle^29^.

Here we report that RAE1 and NUP98 interact with CLOCK and facilitate BMAL1 shuttling. Besides, RAE1 and NUP98 promote the degradation and transcription activation activity of CLOCK: BMAL1 heterodimers. Our current study revealed that RAE1 and NUP98 as the critical elements for BMAL1 shuttling.

## Results

### RAE1 and NUP98 interacts with circadian proteins

**Fig. 1:**
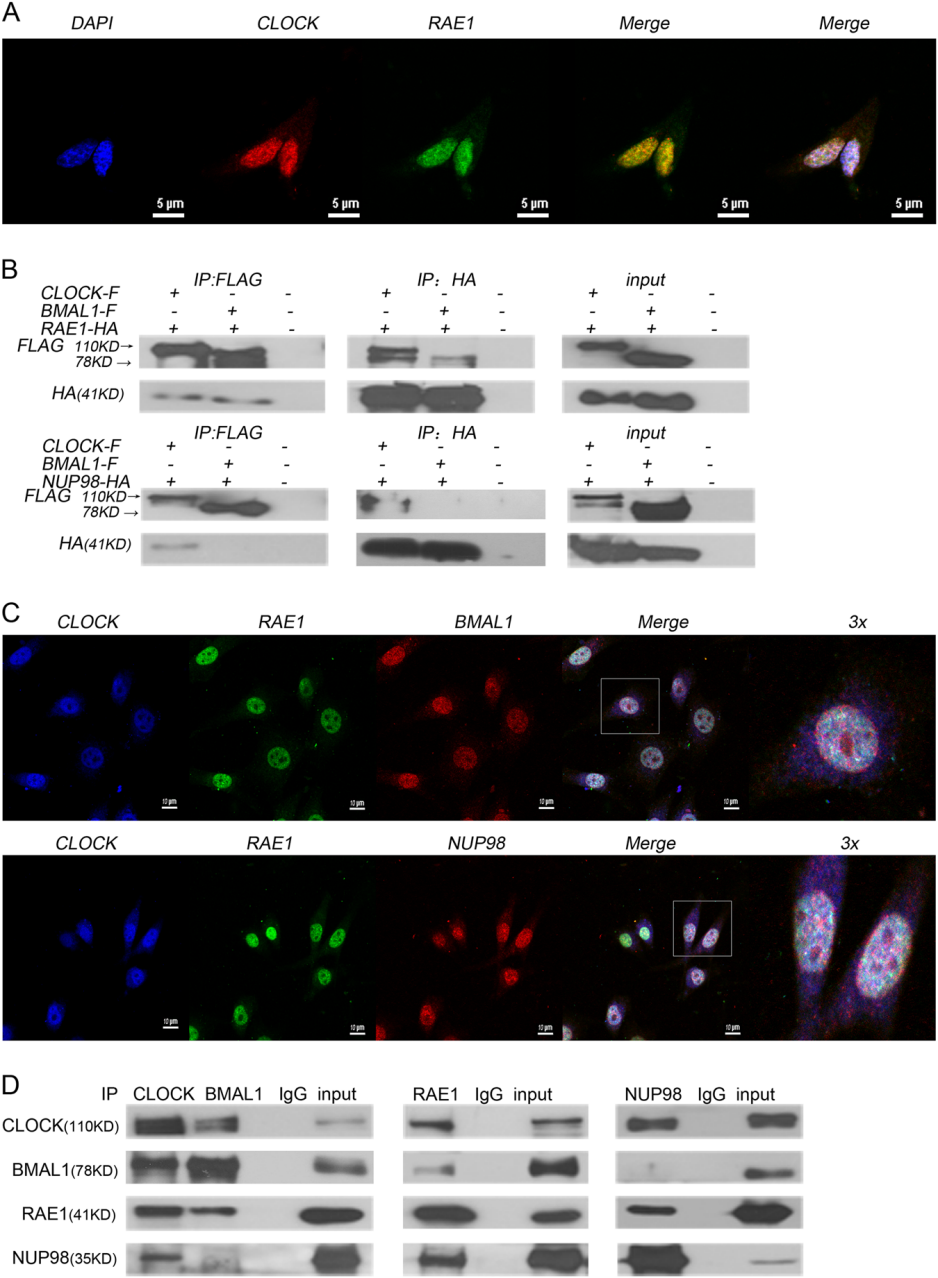
RAE1 and NUP98 interacts with circadian proteins. **a** CLOCK and RAE1 are colocalized in the nucleus of NIH3T3 cells. **b** Exogenous RAE1-HA can directly bind with CLOCK-FLAG and BMAL1-FLAG, but NUP98-HA can only bind with CLOCK-FLAG. **c** CLOCK, RAE1, and BMAL1 are colocalized in the nucleus of NIH3T3 cells; CLOCK, RAE1, and NUP98 are colocalized in the nucleus of NIH3T3 cells. **d** Endogenous RAE1 can directly bind with BMAL1 and CLOCK in NIH3T3 cells, but NUP98 can only bind with CLOCK

To investigate the potential partner of CLOCK protein, we performed a yeast two-hybrid screen using CLOCK PASA domain sequence as a bait (Supplementary 1A) and detected RAE1 as a CLOCK-interacting protein. To confirm the result of Yeast two-hybrid screen, immunofluorescence assays showed that the endogenous RAE1 and CLOCK in NIH3T3 cells were mainly overlapped in the nucleus (Fig. 1a). To further confirm the result, we coexpressed CLOCK-FLAG, BMAL1-FLAG with RAE1-HA, and NUP98-HA in HEK 293T cells, respectively, using anti-FLAG and anti-HA antibodies for immunoprecipitation and immunoblotting. The results showed that exogenous RAE1-HA can directly bind with CLOCK-FLAG and BMAL1-FLAG, but NUP98-HA can only bind with CLOCK-FLAG (Fig. 1b). Immunofluorescence assays showed that the endogenous RAE1, CLOCK, and BMAL1 in NIH3T3 cells were mainly overlapped in the nucleus. RAE1, CLOCK, and NUP98 were also mainly overlapped in the nucleus (Fig. 1c). To further investigate the interaction, NIH3T3 cells, a circadian rhythm model cell line were subjected to endogenous immunoprecipitation. The result showed that endogenous RAE1 can directly bind with BMAL1 and CLOCK in NIH3T3 cells, but NUP98 can only bind with CLOCK (Fig. 1d).

### RAE1 and NUP98 participate in BMAL1 shuttling

**Fig. 2:**
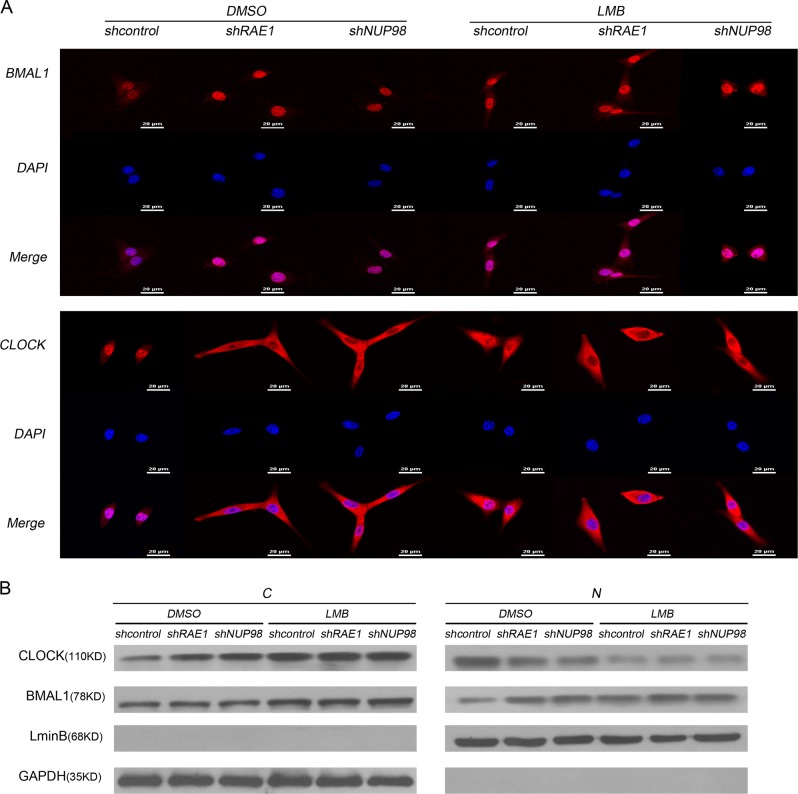
RAE1 or NUP98 knockdown disrupted BMAL1 shuttling and altered the subcellular distribution of CLOCK and BMAL1 in NIH3T3 cells. **a** In RAE1 or NUP98 knocked down NIH3T3 cells, BMAL1 is upregulated, and CLOCK localization is altered from mainly in the nucleus to mainly in the cytoplasm. And NIH3T3 cells treated with LMB showed similar BMAL1 and CLOCK distribution. **b** Cytosolic (C)/nuclear (N) fractionation assay showed that more CLOCK is translocated to the cytoplasm, while more BMAL1 is translocated to the nucleus in RAE1 or NUP98 knocked down NIH3T3 cells. Lamin B1 and GAPDH were employed as internal controls

Our immunofluorescence assays showed that RAE1 or NUP98 knockdown increased the expression of BMAL1 mainly in the nucleus but substantially increased cytoplasmic accumulation and was slightly downregulated in the nucleus of CLOCK (Fig. 2a, Supplementary 1B), suggesting that RAE1 and NUP98 can regulate BMAL1 shuttling. Previous findings suggested that the nuclear export of BMAL1 requires CRM1, and this shuttling can be efficiently blocked by the fungal antibiotic, LMB, by binding to CRM1^14^. To further confirm that RAE1 and NUP98 participate in BMAL1 shuttling, NIH3T3 cells were treated with 10 ng/ml LMB for 6 h. The results showed that RAE1 or NUP98 knockdown and LMB-treated cells have similar BMAL1 and CLOCK distribution (Fig. 2a), suggesting that RAE1 and NUP98 participate in BMAL1 shuttling. The cytosolic (C)/nuclear (N) fractionation assays further confirmed that more CLOCK is translocated to cytoplasm, while more BMAL1 is translocated to nucleus in RAE1 or NUP98 knocked down NIH3T3 cells (Fig. 2b, Supplementary 1C–F).

### RAE1 and NUP98 decrease the CLOCK and BMAL1 protein levels

**Fig. 3:**
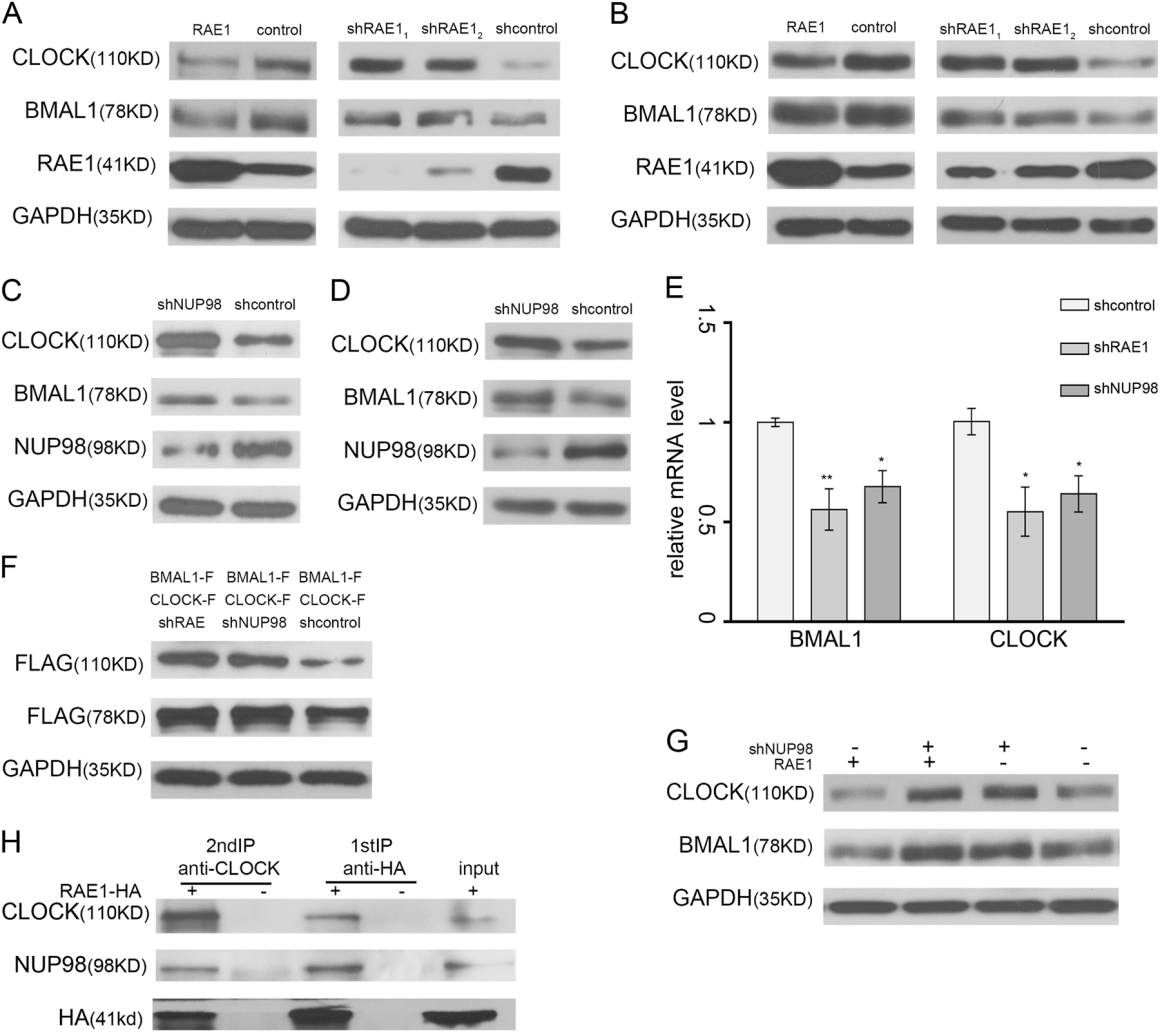
RAE1 and NUP98 decrease CLOCK and BMAL1 protein levels. **a** RAE1 decreases CLOCK and BMAL1 protein levels in HEK293 cells. **b** RAE1 decreases CLOCK and BMAL1 protein levels in NIH3T3 cells. **c** NUP98 knockdown increased CLOCK and BMAL1 protein levels in HEK293 cells. **d** NUP98 knockdown increased CLOCK and BMAL1 protein levels in NIH3T3 cells. **e** Real-time PCR confirmed that the CLOCK and BMAL1 mRNA level are downregulated after RAE1 and NUP98 knockdown. **f** RAE1 or NUP98 knockdown increased exogenous CLOCK-FLAG and BMAL1-FLAG protein levels in HEK293 cells. **g** RAE1 overexpression cannot recover CLOCK and BMAL1 protein levels increased by NUP98 knockdown in NIH3T3 cells. **h** A two-step immunoprecipitation assay showed the existence of a complex comprising RAE1, NUP98 and CLOCK. *P < 0.05; **P < 0.01

Western blot analysis showed that the CLOCK and BMAL1 protein levels was downregulated by overexpression of RAE1 in HEK393 cells, while knockdown of RAE1 led to significant upregulation of CLOCK and BMAL1 protein levels (Fig. 3a). The same results were detected in NIH3T3 cells (Fig. 3b). Since shRAE1_1_ performed better, we chose it for the following experiments. Similarly, NUP98 was knocked down both in HEK393 cells (Fig. 3c) and in NIH3T3 cells (Fig. 3d), and western blot analysis showed that the CLOCK and BMAL1 protein levels were also upregulated. However, quantitative PCR was performed and showed that knockdown of RAE1 or NUP98 suppressed transcription levels of CLOCK and BMAL1 in NIH3T3 cells (Fig. 3e). Furthermore, western blot results showed that knockdown of RAE1 or NUP98 can increase the level of exogenous CLOCK and BMAL1 in HEK393 cells transfected with FLAG-tagged CLOCK and BMAL1, indicating that RAE1 and NUP98 can regulate CLOCK and BMAL1 at the protein level (Fig. 3f). As shown in Fig. 3g, co-transfected RAE1 and shNUP98 have no significant difference with the shNUP98 group. Immunoprecipitation experiments also revealed that CLOCK protein can interact with RAE1 and NUP98 (Fig. 3h). Taking together, RAE1 and NUP98 are working together to regulate the protein level of CLOCK and BMAL1.

### RAE1 and NUP98 enhance BMAL1 shuttling-induced ubiquitination and degradation of BMAL1 and CLOCK

**Fig. 4:**
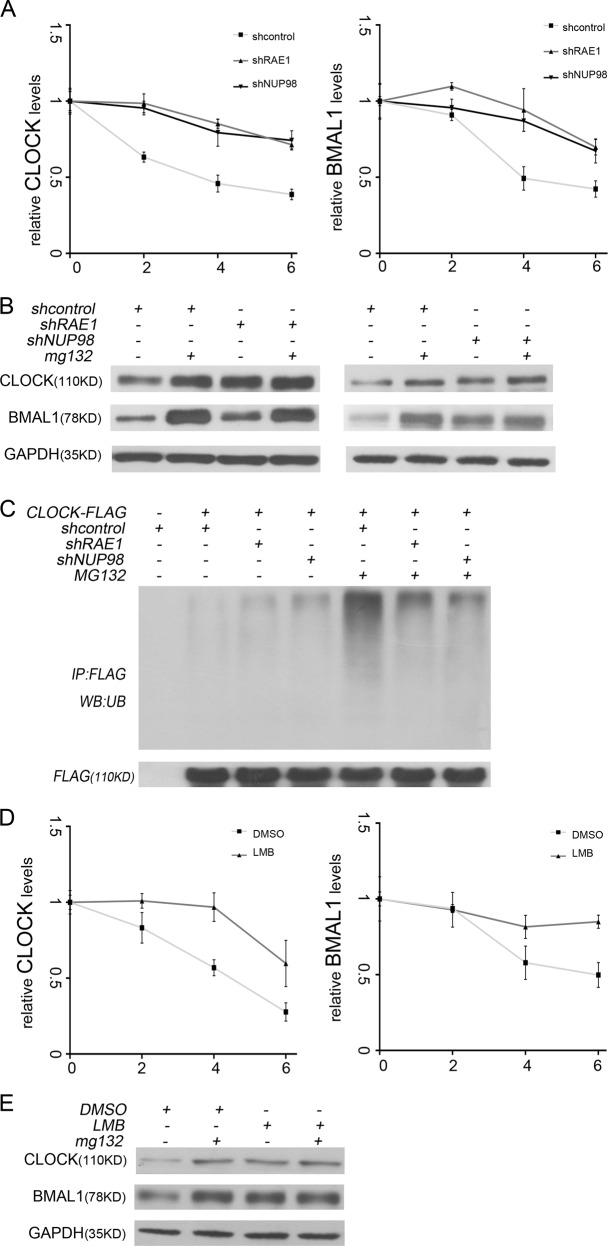
RAE1 and NUP98 enhance ubiquitination and degradation of BMAL1 and CLOCK induced by BMAL1 shuttling. **a** RAE1 and NUP98 knockdown suppressed degradation of BMAL1 and CLOCK in NIH3T3 cells. **b** The knockdown of RAE1 and NUP98 regulated the protein levels of CLOCK and BMAL1 through proteasome pathway. **c** Ubiquitination of CLOCK-FLAG is downregulated by RAE1 or NUP98 knockdown. **d** LMB treatment suppressed degradation of BMAL1 and CLOCK in NIH3T3 cells. **e** MG132 treatment can recover BMAL1 and CLOCK expression suppressed by LMB treatment

As shown above, these results indicate that RAE1 and NUP98 participate in BMAL1 shuttling and decreases CLOCK and BMAL1 protein levels. To examine whether RAE1 and NUP98 reduce the stability of CLOCK and BMAL1, NIH3T3 cells were treated with 50 µg/ml cycloheximide (CHX) to inhibit protein synthesis and harvested for western blot analysis. The results showed that RAE1 or NUP98 knockdown decreased the degradation of CLOCK and BMAL1 (Fig. 4a, Supplementary 2A). In NIH3T3 cells treated with proteasome inhibitor 20 µM MG132 for 6 h, RAE1 or NUP98 knockdown cannot influence CLOCK and BMAL1 protein levels, suggesting that RAE1 and NUP98 knockdown can inhibit proteasomal degradation of CLOCK and BMAL1 (Fig. 4b). Additionally, HEK293 cells treated with 20 µM MG132 for 6 h were co-transfected with CLOCK-FLAG and shRAE1 or shNUP98, then harvested for immunoprecipitation with anti-FLAG and anti-ubiquitin. The results showed that RAE1 or NUP98 knockdown can decrease the level of ubiquitination of CLOCK (Fig. 4c). To examine whether the degradation of CLOCK and BMAL1 is associated with BMAL1shuttling, NIH3T3 cells were co-treated with 10 ng/ml LMB and 50 µg/ml CHX. The results showed that LMB can decrease the degradation of CLOCK and BMAL1 (Fig. 4d, Supplementary 2B). In NIH3T3 cells treated with 20 µM MG132 for 6 h, meanwhile 10 ng/ml LMB co-treated for 2 h before harvest, LMB treatment cannot influence CLOCK and BMAL1 protein levels, suggesting that LMB treatment inhibits proteasomal degradation of CLOCK and BMAL1 (Fig. 4e). Taken together, these results suggest that RAE1 and NUP98 can enhance BMAL1 shuttling-induced ubiquitination and degradation of BMAL1 and CLOCK.

### RAE1 and NUP98 promotes the transcriptional activation activity of the CLOCK: BMAL1 heterodimer

**Fig. 5:**
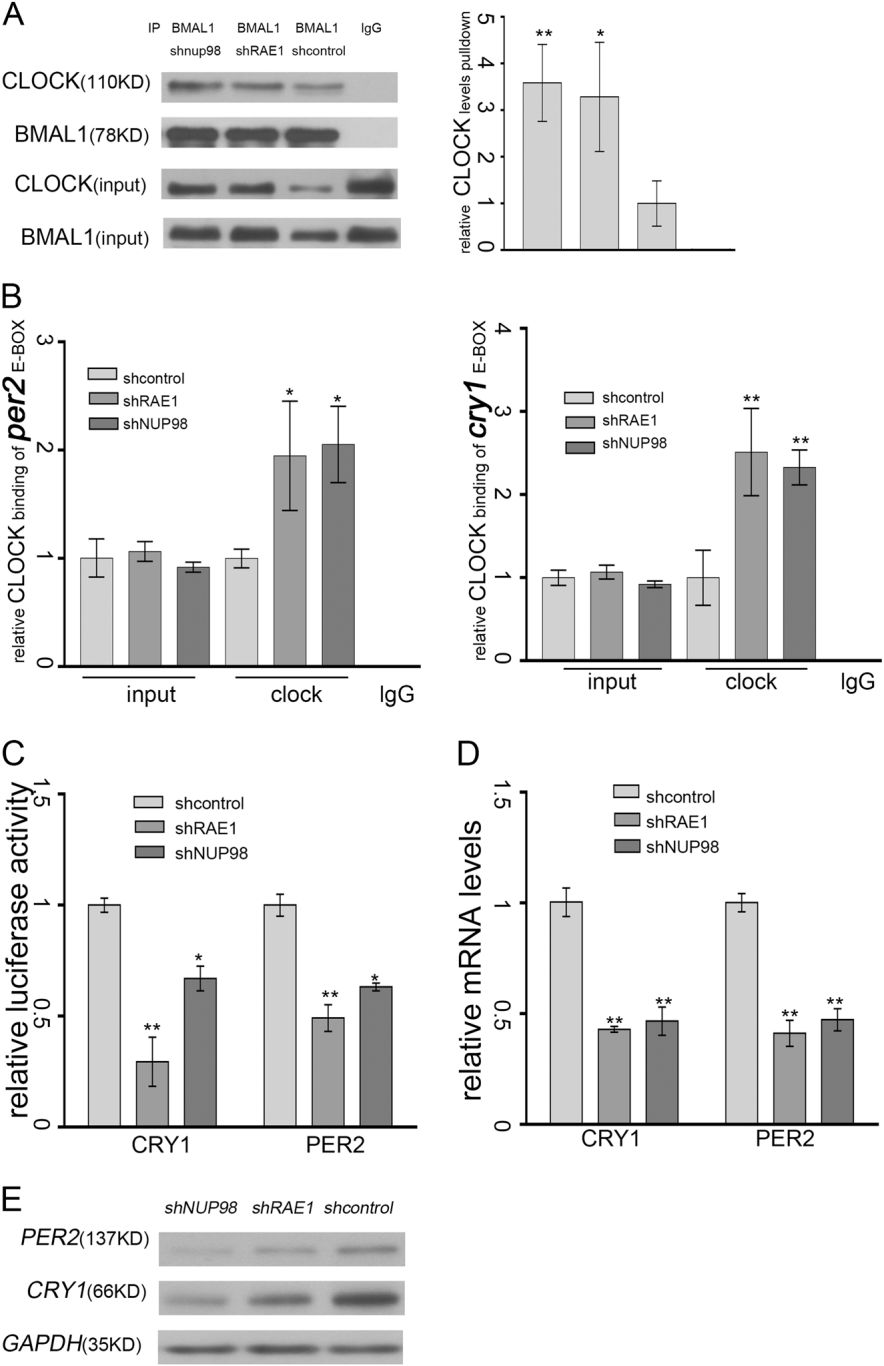
RAE1 and NUP98 promotes the transcriptional activation activity of the CLOCK: BMAL1 heterodimer. **a** RAE1 or NUP98 knockdown enhanced the interaction of BMAL1 and CLOCK in the nucleus. Cells were harvested for Cytosolic (C)/nuclear (N) fractionation assay, and the nucleus lysates were immunoprecipitated by anti-CLOCK antibody. The intensity of bands were quantified using the quantity one software; the value is given for the amount of CLOCK been pulled down. Data are represented as mean ± SD (*P < 0.05; **P < 0.01). **b** Chromatin immunoprecipitation assay tested with quantitative PCR showed that RAE1 and NUP98 knockdown increased the interaction of CLOCK and E-box elements in BMAL1 or CLOCK promoter. Data are represented as mean ± SD (*P < 0.05; **P < 0.01). **c** Knockdown of RAE1 and NUP98 downregulated transcription activity of the CLOCK: BMAL1 heterodimer. Data are represented as mean ± SD (*P < 0.05; **P < 0.01). **d** RAE1 and NUP98 knockdown downregulated PER2 and CRY1 at the mRNA level in NIH3T3 cells. **e** RAE1 and NUP98 knockdown downregulated PER2 and CRY1 at the protein level in NIH3T3 cells

To further investigate the influence of RAE1 and NUP98 to the CLOCK and BMAL1 heterodimer, cytosolic (C)/nuclear (N) fractionation assays were performed in RAE1 or NUP98 knockdown NIH3T3 cells, and the nuclear lysates were used for immunoprecipitates of anti-BMAL1. The results showed that RAE1 and NUP98 knockdown enhanced the interaction between BMAL1 and CLOCK in the nucleus (Fig. 5a). Then we performed chromatin immunoprecipitation (Chip) experiments with anti-CLOCK to explore whether knockdown of RAE1 or NUP98 influence CLOCK and BMAL1 heterodimer binding with the E-box region on PER2 and CRY1 promoters in NIH3T3 cells. Interestingly, quantification with semiquantitative PCR (Supplementary 3A) and real-time PCR (Fig. 5b) showed that knockdown of RAE1 or NUP98 promote CLOCK and BMAL1 heterodimers binding with the E-box regions on promoters of PER2 and CRY1. However, RAE1 and NUP98 cannot bind to CLOCK and BMAL1 (Supplementary 3B). Then transcriptional activity assay were performed, and NIH3T3 cells were transfected with Per2-luciferase reporter or Cry1-luciferase reporter to measure the influence of RAE1 or NUP98 knockdown to the transcription activity of the CLOCK: BMAL1 heterodimer. The results showed that knockdown of RAE1 and NUP98 decreased the transcriptional activity of CLOCK: BMAL1 heterodimer (Fig. 5c). Furthermore, western blot analysis (Fig. 5d) and quantitative PCR (Fig. 5e) was performed and showed that knockdown of RAE1 and NUP98 suppressed PER2 and CRY1 expression at both the protein levels and transcription levels in NIH3T3 cells.

### RAE1 enhances the amplitude of PER2 and CRY1 expression

**Fig. 6:**
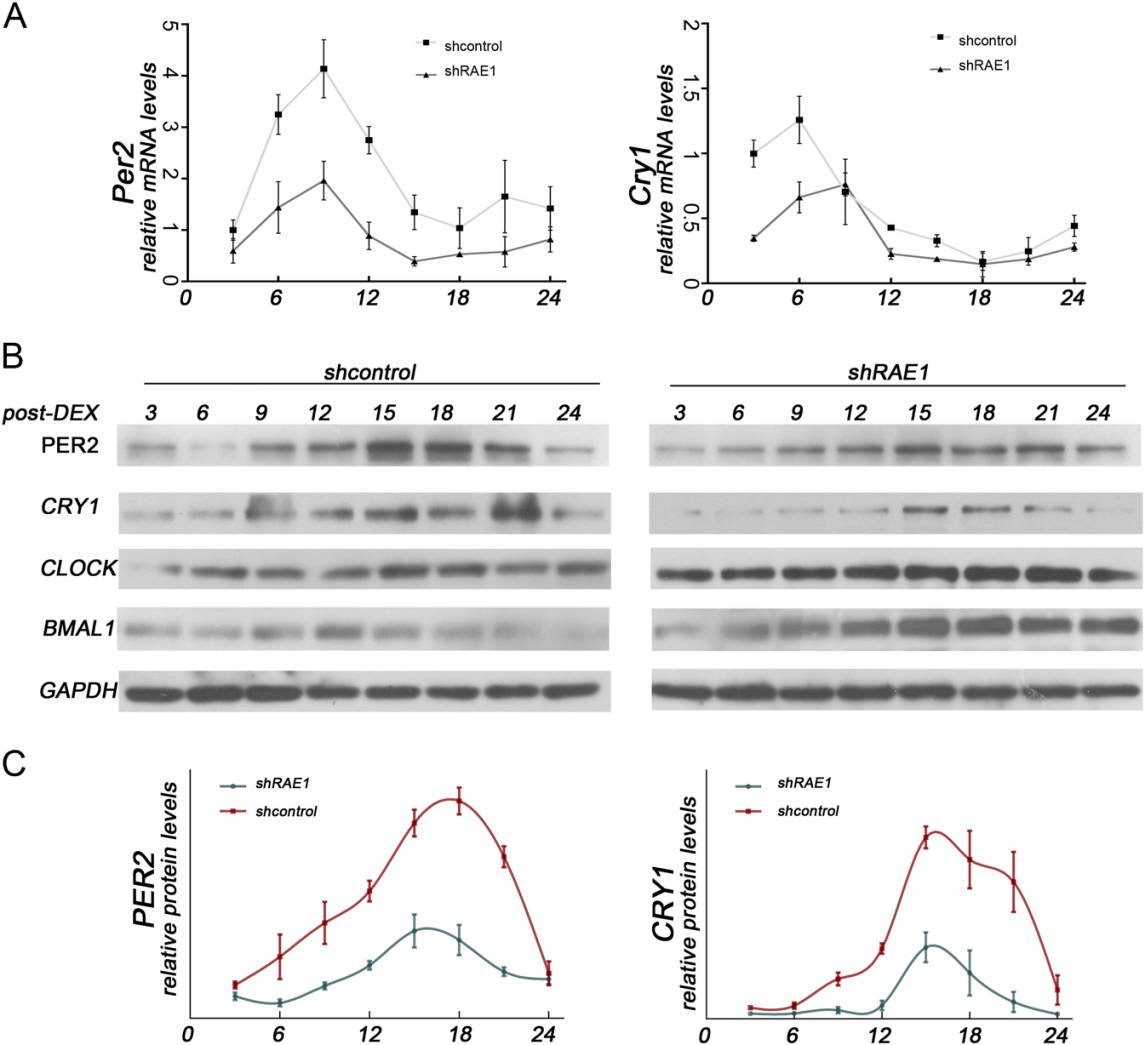
RAE1 enhances the amplitude of PER2 and CRY1 expression. **a** NIH3T3 cells transfected with shRAE1 or shcontrol were synchronized by treatment with 100 nM dexamethasone (DEX) for 2 h. Total lysates were prepared at the indicated times post-synchronization. The mRNA levels of *Per2* and *Cry1* were detected with quantitative PCR. **b**, **c** The protein levels of PER2 and CRY1 were detected with western blot at the indicated times post-synchronization. The intensity of bands were quantified using the quantity one software; the value is given for the amount of CRY1 and PER2 present in NIH3T3 cells at different times. Data are represented as mean ± SD

To explore the function of RAE1 on the cyclic expression of clock genes, NIH3T3 cells transfected with shRAE1 or shcontrol were synchronized by dexamethasone (DEX) 100 nM for 2 h. The mRNA levels of PER2, CRY1, Dbp, and Rev-Erb at the indicated time post-synchronization were detected with quantitative PCR (Fig. 6a, Supplementary 3C). The protein levels of PER2 and CRY1 at the indicated times post-synchronization were detected with western blot and then quantified using the quantity one software (Fig. 6b, c). The results showed that BMAL1, PER2, and CRY1 protein expression are roughly 24-h oscillated in the shcontrol group, but the amplitude of CCGs are distinctly reduced in the shRAE1 group.

## Discussion

The heterodimeric transcription factor CLOCK: BMAL1 are the key components in circadian rhythms by occupying the positive limb of the transcription–translation feedback loop^3,4^. Previous studies have shown that BMAL1 is crucial for translocation of CLOCK: BMAL1 heterodimer^14,20^. When BMAL1 is absent or mutated, CLOCK failed to accumulate to nucleus and was found mainly in the cytoplasm^14,30^, and BMAL1 shuttling is CRM1 dependent^14,31^. However, factors that regulate BMAL1 shuttling remain largely unclear.

Our present research showed that RAE1 can interact with BMAL1 and CLOCK, while NUP98 can only bind with CLOCK (Fig. 1b, d). Immunofluorescence assays showed that RAE1, NUP98, and CLOCK mainly overlapped in the nucleus, while RAE1, BMAL1, and CLOCK also overlapped in the nucleus (Fig. 1c). Knockdown of RAE1 and NUP98 lead to accumulation of BMAL1 in the nucleus and CLOCK in the cytoplasm (Fig. 2a, b). However, RAE1 and NUP98 knockdown cannot regulate BMAL1 and CLOCK protein distribution in the CRM1 inhibitor LMB-treated cells (Fig. 2a, b), suggesting that RAE1 and NUP98 participate in BMAL1 shuttling, especially regulating the BMAL1 export from the nucleus. Besides, RAE1 and NUP98 knockdown increase CLOCK and BMAL1 protein levels through suppressing their ubiquitination and degradation, which can be suppressed by inhibitor LMB (Figs. 3 and 4).

Notably, RAE1 and NUP98 knockdown also increase the interaction of CLOCK and BMAL1 in the nucleus (Figure 5a). Meanwhile, Chip assays showed that RAE1 and NUP98 knockdown promote CLOCK binding to the E-box region in the promoters of PER2 and CRY1 (Fig. 5b, c). However, the transcriptional activation activity of the CLOCK: BMAL1 heterodimer decreased (Fig. 5d), consistent with the decreased expression of PER2 and CRY1 (Fig. 5e, f). Our above results indicated that RAE1 and NUP98 can promote the shuttling of BMAL1 and further regulate the degradation of CLOCK and BMAL1 and the transcriptional activation activity of the heterodimer. This finding is consistent with previous report that BMAL1 shuttling promotes nuclear translocation of CLOCK and E-box-dependent clock gene transcription, coupled with rapid degradation of BMAL1 and CLOCK^14^. Subsequently, RAE1 knockdown suppressed the amplitude of PER2 and CRY1 protein levels (Fig. 6).

**Fig. 7:**
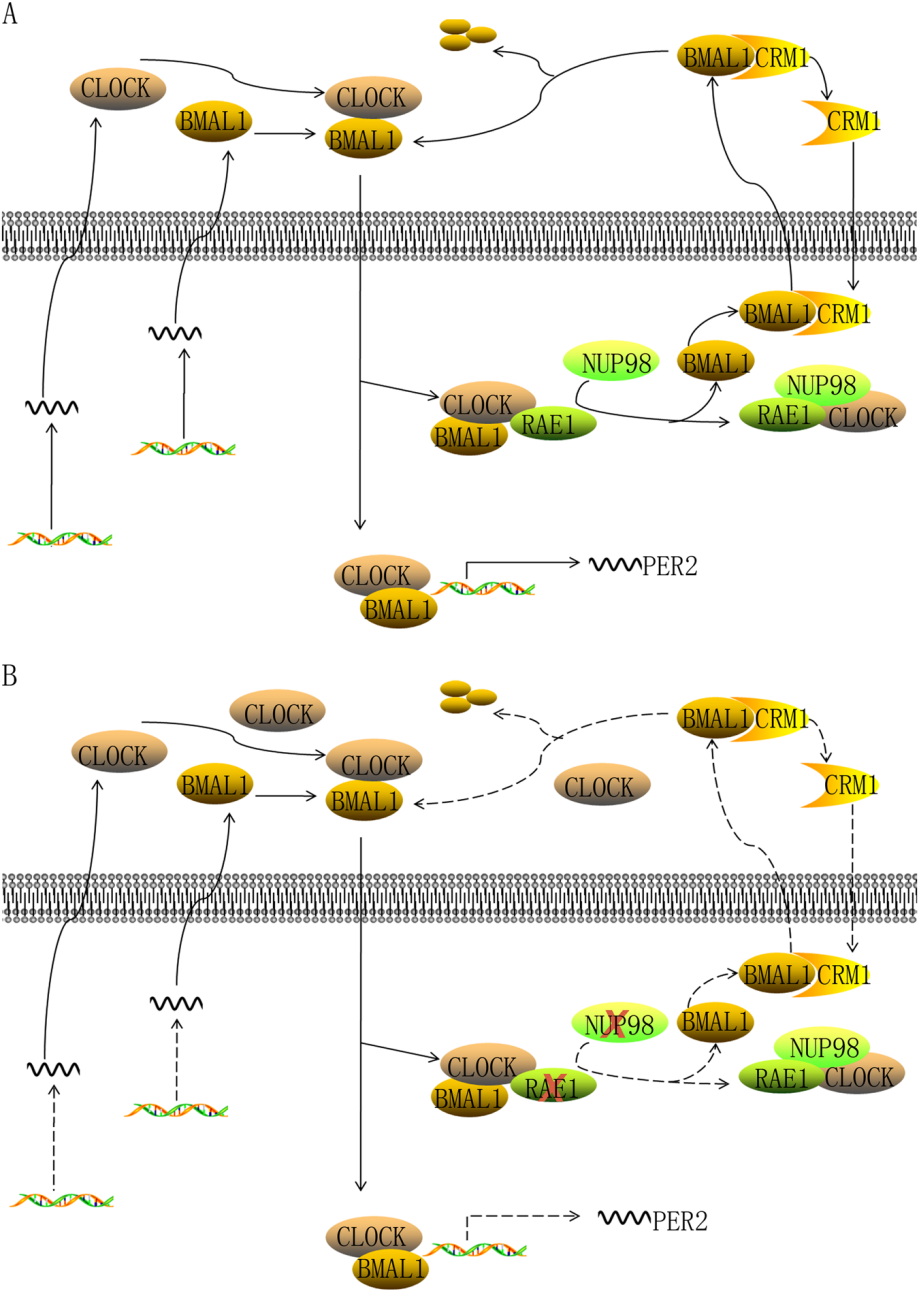
Working model of the RAE1 and NUP98 in circadian clock. **a** RAE1 and NUP98 participate inBMAL1 shuttling and promote the degradation and activity of BMAL1 and CLOCK. **b** When cells absented RAE1 or NUP98

Taken together, our findings suppose a hypothetical mechanism that RAE1 regulates CLOCK and BMAL1 (Fig. 7). RAE1 binds to CLOCK: BMAL1 heterodimer in the nucleus and then NUP98 binds to RAE1 that already interact with the heterodimer, releasing BMAL1 from the complex for CRM1-dependent exporting to cytoplasm to expedite BMAL1 shuttling. And the RAE1-induced BMAL1 shuttling is crucial for CLOCK entering nucleus and influences their degradation and transcriptional activation activity (Fig. 7a). In contrast, RAE1 and NUP98 knockdown suppress BMAL1 exporting to cytoplasm, leading to nuclear accumulation and transcriptional inhibition of CLOCK: BMAL1 heterodimer (Fig. 7b).

In conclusion, our work described a novel function of RAE1 and NUP98 regulating BMAL shuttling, providing a novel insight into the molecular mechanism of RAE1 in circadian clock.

## Materials and methods

### Yeast two-hybrid screening

pGBKT7-PASA was used as bait plasmid and a commercial human testis cDNA library (Clontech) as prey library. The experimental procedures reference manuals of the manufacturer (Clontech). Hybrid diploid was screened by growth on SD/-Trp/-Leu/-His/-Ade plates added with X-α-gal and Aureobasidin A. Blue colonies were considered as positive clones. The prey plasmids were rescued from the positive colonies by transformation into *Escherichia coli*. The cDNA fragments of the positive clones were sequenced and aligned with sequences from the NCBI database.

### Cell culture

Cell line of NIH-3T3 fibroblasts and HEK 293 were maintained in State Key Laboratory of Biotherapy of West China Hospital. All cells were cultured in Dulbecco modified Eagle medium supplemented with 10% fetal bovine serum, 100 U/ml penicillin, and 100 µg/ml streptomycin, under 5% CO_2_ at 37 °C. The transfection was performed with jetPRIME^TM^ (Polyplus-transfection, SA, France) according to the manufacturer’s protocol; 48 h after transfection, cells were pretreated with CHX (50 µg/ml), MG132 (20 µM), LMB (10 ng/ml), or DEX (100 nM) for the indicated times; and they were purchased from Sigma (St. Louis, MO, USA). Cells were harvested and analyzed by western blotting using appropriate antibodies. All following experiments were repeated at least three times unless stated otherwise.

### Cytosolic (C)/nuclear (N) fractionation

To isolate the cytoplasmic component from the nuclear one, cells were treated with the cytosolic (C)/nuclear (N) fractionation extraction kit (Beyotime Biotechnology, Shanghai, China) according to the manufacturer’s instructions.

### Plasmids and antibodies

CDS encoding RAE1 and NUP98 were synthesized and inserted into pcDNA3.1 vector. CDS encoding CLOCK and BMAL1 were synthesized and inserted into pHBLV-CMVIE-ZsGreen-Puro vector bought from Hanbio company (Shanghai, China), shRNA against RAE1 and NUP98 were synthesized and inserted into plKO.1, and the target sequences of these shRNA were as follows:

Human RAE1 shRNA (shRAE11): 5′-TGGGATACTCGATCGTCAAAT-3′

Human RAE1 shRNA (shRAE12): 5′-CCAGAGTTGTTTCTCTCCACT-3′

Human NUP98 shRNA (shNUP98): 5′-CCCTTGCAGATGGCTCTTAAT-3′

Murine RAE1 shRNA (shRAE11): 5′-CTGGGACTGTTGGAGTTTCAT-3′

Murine RAE1 shRNA (shRAE12): 5′-CCACAATCCAATGAAGGATAT-3′

Murine NUP98 shRNA (shNUP98): 5′-GCCCTGACTTTAGGAACCAATA-3′

Antibodies against each named protein were: CLOCK for Immunofluorescence staining (Santa Cruz, CA, USA); CLOCK for western blotting (Cell signaling technology, MA, USA); CLOCK for Chip and coimmunoprecipitation (Abcam, MA, USA); BMAL1for immunofluorescence staining (Santa Cruz, CA, USA); BMAL1 for western blotting (Cell signaling technology, MA, USA); GAPDH (Abcam, MA, USA); HA, FLAG (Zen Bioscience, CD, China); CRY1 (Santa Cruz, CA, USA); PER2 (Santa Cruz, CA, USA); RAE1 (Abcam, MA, USA); and NUP98 (Abcam, MA, USA).

### Immunofluorescence

Cells were fixed with 4% paraformaldehyde in phosphate-buffered saline (PBS) for 10 min and permeabilized with 0.5% Triton X-100 for 5 min, blocked with 1% bovine serum albumin for 30 min, and incubated overnight at 4 °C with primary antibody. The hosts of primary antibodies are CLOCK(goat), RAE1(rabbit), BMAL1(mouse), and NUP98(mouse) and finally incubated with Alexa Fluor® 488 (Thermo Fisher Scientific, CA, USA), Alexa Fluor® 555 (Thermo Fisher Scientific, CA, USA), Alexa Fluor® 647 (Thermo Fisher Scientific, CA, USA) for 1 h at room temperature. Each step was followed by two 5-min washes in PBS. The nucleus was stained with 4,6-diamidino-2-phenylindole (Sigma Aldrich, St. Louis, MO, USA) for 3 min. Fluorescent images were obtained with a confocal microscope (Nikon, Tokyo, Japan).

### Coimmunoprecipitation and western blotting

For coimmunoprecipitation and western blotting, cells were lysed after transfecting with the designated plasmids in universal protein extraction buffers (Bioteke, Peking, China) containing protease inhibitor (Roche, Basel, Switzerland). Extracted proteins were immunoprecipitated with special antibody and protein A+G agarose beads (Beyotime Biotechnology, Shanghai, China). Bound proteins were separated using sodium dodecyl sulfate (SDS)–polyacrylamide gel electrophoresis, transferred to polyvinylidene difluoride membranes (Millipore, Billerica, MA, USA), and detected with specific appropriate primary antibodies and horseradish peroxidase-conjugated secondary antibodies. Specific proteins were visualized using an enhanced chemiluminescence (ECL) western blotting detection system (Millipore, Billerica, MA, USA).

### Chip and real-time PCR

Chip assay was performed using a Chip Assay Kit (Beyotime Biotechnology, Shanghai, China) according to the manufacturer’s directions. Cells were formalin fixed for 10 min, then crosslinking was stopped by adding 125 mM glycine. Lysed with SDS lysis buffer containing protease inhibitor cocktail (Roche, Basel, Switzerland); cell lysates were ultra-sonicated by an Ultrasonic cell lyser, and immunoprecipitated with 2 µg antibodies. Eluted and purified DNA was subjected to PCR, using primers as follows:

PER2-S: GGACGACGGGTAGCACGAA

PER2-A: GCCGCTGTCACATAGTGGAAAA

CRY1-S: CATAGAGGCAGGAAGGAGAA

CRY1-A: ATCAGCCTTTCTTTGGTTCT

Quantitative PCR was performed in a RT-qPCR (CFX96 Touch, Bio-Rad Laboratories, Inc) with a denaturation step at 94 °C for 10 min, followed by 45 cycles of denaturation at 94 °C for 20 s, annealing at 60 °C for 30 s, and extension at 72 °C for 40 s using the BIO-RAD SYBR (Bio-Rad Laboratories, CA, USA). Semiquantitative PCR was performed for only 22 cycles. PCR was performed using primers as follows:

GAPDH-S: AAGGTGAAGGTCGGAGTCAA

GAPDH-A: AATGAAGGGGTCATTGATGG

CLOCK-S: CCTGAGACAGCTGCTGACAA

CLOCK-A: TGGTTGGTGTTGAGGAAGGG

BMAL-S: AGTCTGTCTTCAAGATCCTCAACTAC

BMAL-A: CTGGAAGTCCAGTTTTTGCATCTATG

CRY1-S: CTCCTCCAATGTGGGCATCAA

CRY1-A: CCACGAATCACAAACAGACGG

PER2-S: GGTGCACAGCCCTCATTCTTTTCA

PER2-A: CCTCACTTTTCCCCAAGTGTCCAA

DBP-S: GGAACTGAAGCCTCAACCAATC

DBP-A: CTCCGGCTCCAGTACTTCTCA

REV-ERBΑ-S: CCCTGGACTCCAATAACAACACA

REV-ERBΑ-A: GCCATTGGAGCTGTCACTGTAG

### Dual luciferase assay

For luciferase analysis, sequences of E-box region and flanking 6 bp at each side were cloned into pGL3 reporter vector. In all, 100 ng plasmid DNA and 100 ng renilla control plasmid were co-transfected into HeLa cells. Dual luciferase activity assays were performed 48 h after transfection according to the manufacturer’s directions (Promega, Madison, WI, USA).

### Statistical analysis

All experiments were repeated at least three times unless stated otherwise. Western blot results were quantified using the quantity one software. GraphPad software were employed to determine the rhythmically express of proteins. Differences between experimental groups were determined using Student’s *t* test. Statistical significance was accepted when *P* < 0.05.

## Supplementary information

Supplementary 1

Supplementary 2

Supplementary 3
