# Supplementary figures and images for: RAE1 promotes BMAL1 shuttling and regulates degradation and activity of CLOCK: BMAL1 heterodimer
Source: Cell Death Dis. 2019 Jan 25;10(2):62. doi: 10.1038/s41419-019-1346-2 (PMC6347605; doi:10.1038/s41419-019-1346-2)

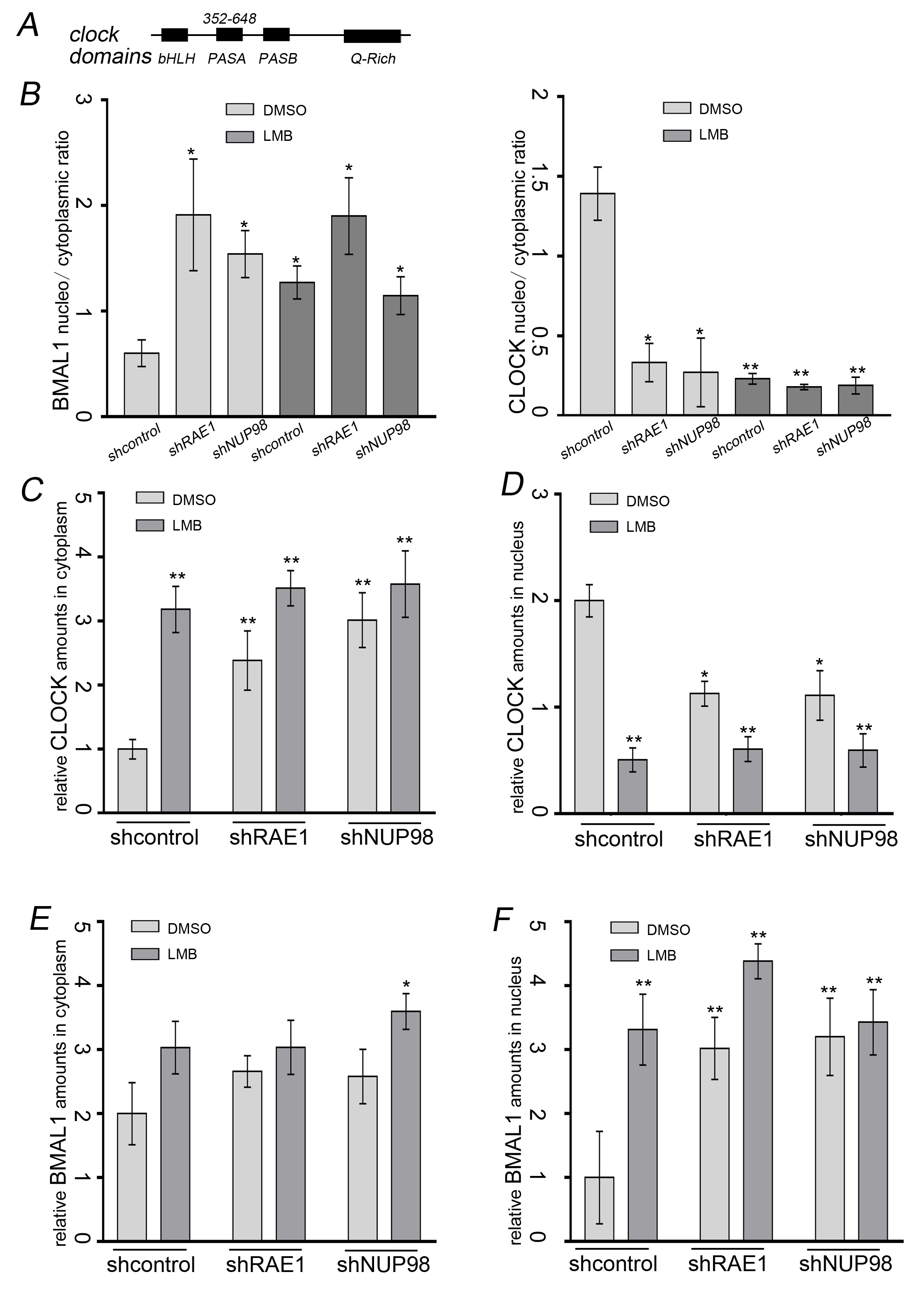

Supplement: Supplementary file 1 — Supplementary 1 [file 41419_2019_1346_MOESM1_ESM.tif]

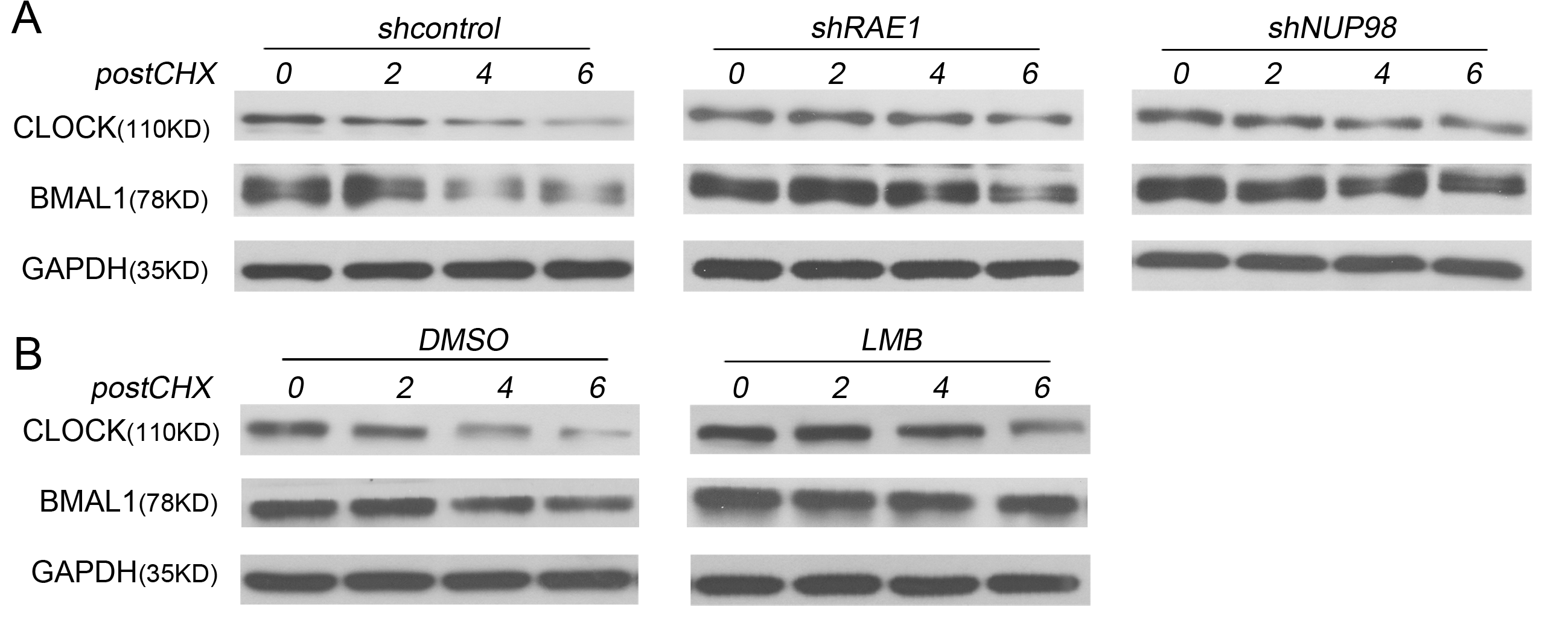

Supplement: Supplementary file 2 — Supplementary 2 [file 41419_2019_1346_MOESM2_ESM.tif]

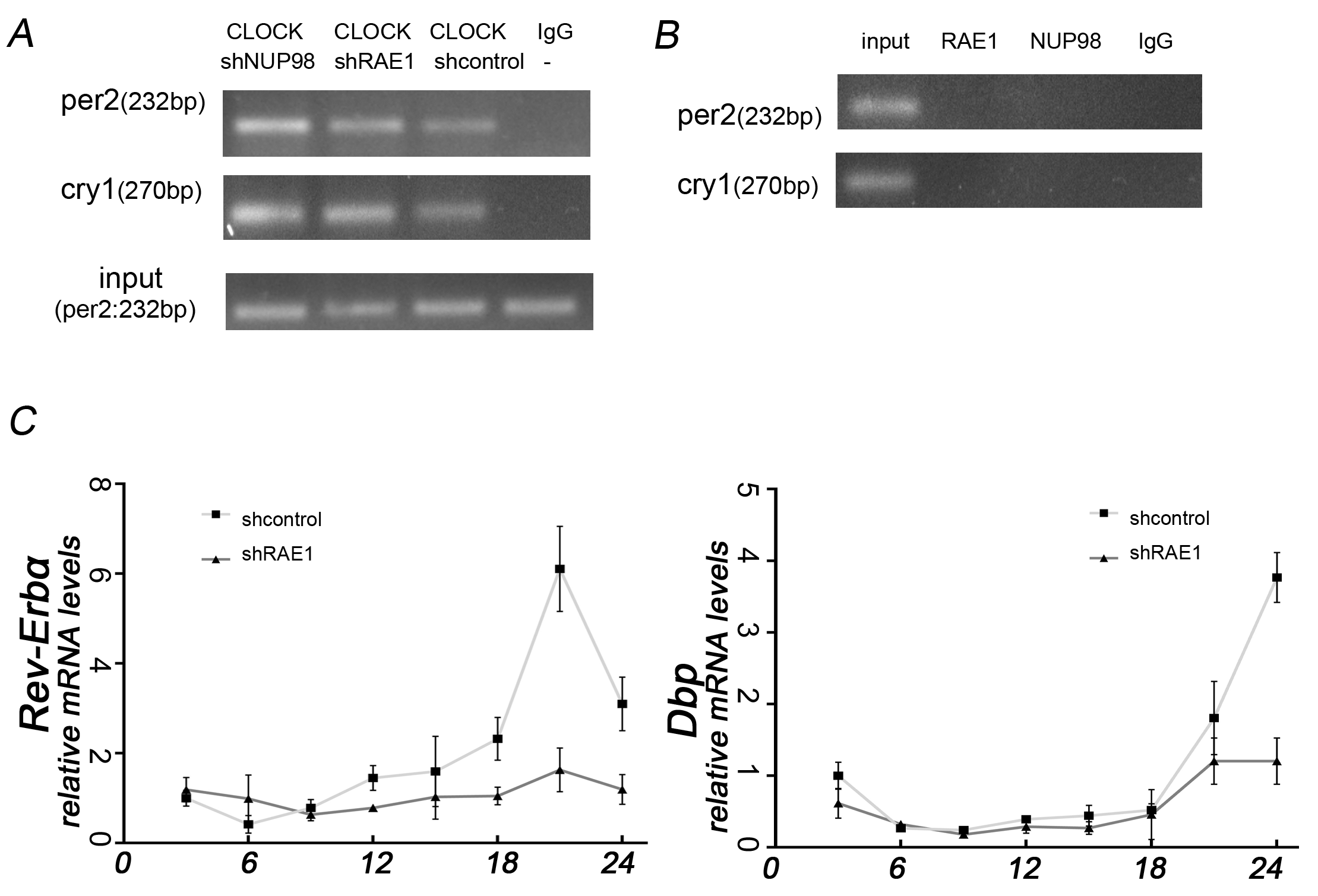

Supplement: Supplementary file 3 — Supplementary 3 [file 41419_2019_1346_MOESM3_ESM.tif]
